# Electrical Stimulation of Neurons with Quantum Dots via Near-Infrared Light

**DOI:** 10.1021/acsnano.2c01989

**Published:** 2022-05-02

**Authors:** Onuralp Karatum, Humeyra Nur Kaleli, Guncem Ozgun Eren, Afsun Sahin, Sedat Nizamoglu

**Affiliations:** † Department of Electrical and Electronics Engineering, Koc University, Istanbul 34450, Turkey; ‡ Research Center for Translational Medicine, Koc University, Istanbul 34450, Turkey; § Department of Biomedical Science and Engineering, Koc University, Istanbul 34450, Turkey; ∥ Department of Ophthalmology, Medical School, Koc University, Istanbul 34450, Turkey

**Keywords:** near-infrared, neural stimulation, optical stimulation, quantum dot, photovoltaic, electrical stimulation

## Abstract

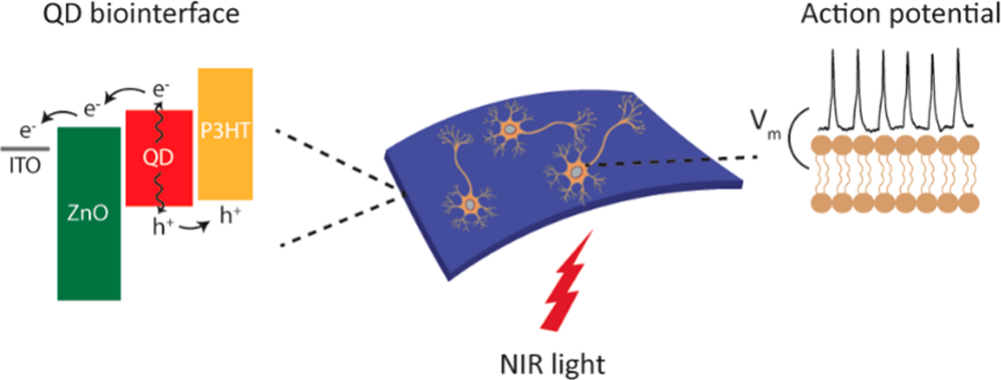

Photovoltaic biointerfaces offer wireless and battery-free bioelectronic medicine via photomodulation of neurons. Near-infrared (NIR) light enables communication with neurons inside the deep tissue and application of high photon flux within the ocular safety limit of light exposure. For that, nonsilicon biointerfaces are highly demanded for thin and flexible operation. Here, we devised a flexible quantum dot (QD)-based photovoltaic biointerface that stimulates cells within the spectral tissue transparency window by using NIR light (λ = 780 nm). Integration of an ultrathin QD layer of 25 nm into a multilayered photovoltaic architecture enables transduction of NIR light to safe capacitive ionic currents that leads to reproducible action potentials on primary hippocampal neurons with high success rates. The biointerfaces exhibit low in vitro toxicity and robust photoelectrical performance under different stability tests. Our findings show that colloidal quantum dots can be used in wireless bioelectronic medicine for brain, heart, and retina.

## Introduction

Optical control of neural activity offers real-time interrogation of neural networks and minimally invasive treatment of neural system diseases.^1−3^ In the optical spectrum, near-infrared (NIR) light advantageously allows a higher penetration depth into the body owing to the marginal tissue absorption and scattering,^4,5^ removes the implantation requirement of electrical or optical signal delivery components into tissue, and enables application of a higher photon flux within safe light exposure limits because of lower photon energies compared to visible light. For example, upconversion nanoparticles (NPs) recently rendered NIR sensitivity to optogenetic systems operating in the visible spectrum, and overcame the light penetration limitation of visible light and implantation requirement of light-delivery fibers into the tissue.^6^ Because of these benefits, there is an increasing trend for developing NIR-sensitive nanoparticles, devices, and systems that operate in the tissue transparency window to control neural activity.^7−11^

Bioelectronic medicine enables treatment of diseases via stimulation of cells without any drug delivery or genetic variation of native tissue. Among different device configurations, photovoltaic biointerfaces advantageously offer a wireless and battery-free neurostimulation tool that removes the need for wires, which leads to surgical complications, and replacement of the battery. For example, silicon photovoltaic biointerfaces enable shorter and simpler surgical procedures for retinal implants, and they convert NIR light to ionic currents for stimulation of the tissue, which have enabled successful clinical outcomes to recover vision against blindness due to age-macular degeneration.^12,13^ However, the low absorption coefficient of silicon at NIR (383 cm^–1^ at 880 nm) necessitates a 30 μm-thick and rigid photoactive layer.^14^ Thin and flexible optoelectronic devices can be a better alternative to fit into the curvature of tissue. For example, recently organic pigments and polymers as photoactive layers enabled flexible photocapacitors as cuff electrodes on peripheral nerves and implants fitting to the curvature of the retina, respectively.^15,16^

Alternatively, colloidal quantum dots (QDs) have a unique size tunable bandgap via a quantum confinement effect, solution-processable fabrication, and a high absorption coefficient for thin photoactive layers.^17^ In addition, they have high optical stability with minimal photobleaching or chemical degradation.^18^ So far, core or core/shell structures of different QDs like mercury telluride (HgTe), cadmium selenide (CdSe), indium phosphide (InP), and aluminum antimonide (AlSb) were successfully used in photovoltaic biointerface architectures for photostimulation of neurons, but their operation was limited within the visible range.^19−23^ Alternatively, lead sulfide (PbS), which has a Bohr exciton radius of 18 nm and bulk bandgap of 0.41 eV, enables sensitive tuning of the absorption edge within the NIR spectral range.^24^

Here, we developed flexible NIR-sensitive biointerfaces by using quantum dots. Integration of an ultrathin PbS QD layer of 25 nm into a multilayered photovoltaic architecture generates a capacitive photoresponse, which is a safe charge injection mechanism for extracellular neurostimulation. The charge injection density of the biointerfaces was significantly enhanced by modifying the return electrode with a supercapacitor ruthenium dioxide (RuO_2_) coating. Efficient photoconversion in physiological medium leads to generation of temporally precise action potentials in hippocampal neurons under 780 nm photoexcitation with more than 80% success rates up to 20 Hz stimulation frequency within the ocular safety limits. The biointerfaces are resistant to various stress tests and chronic photoexcitation and show low cytotoxicity for in vitro hippocampal neuron cultures. Altogether, the NIR-sensitive QD-based biointerface architecture presented herein has great potential for building minimally invasive neurostimulators for performing brain, cardiac, and retinal stimulation.

## Results and Discussion

### Biointerface Design and Operation

**Figure 1:**
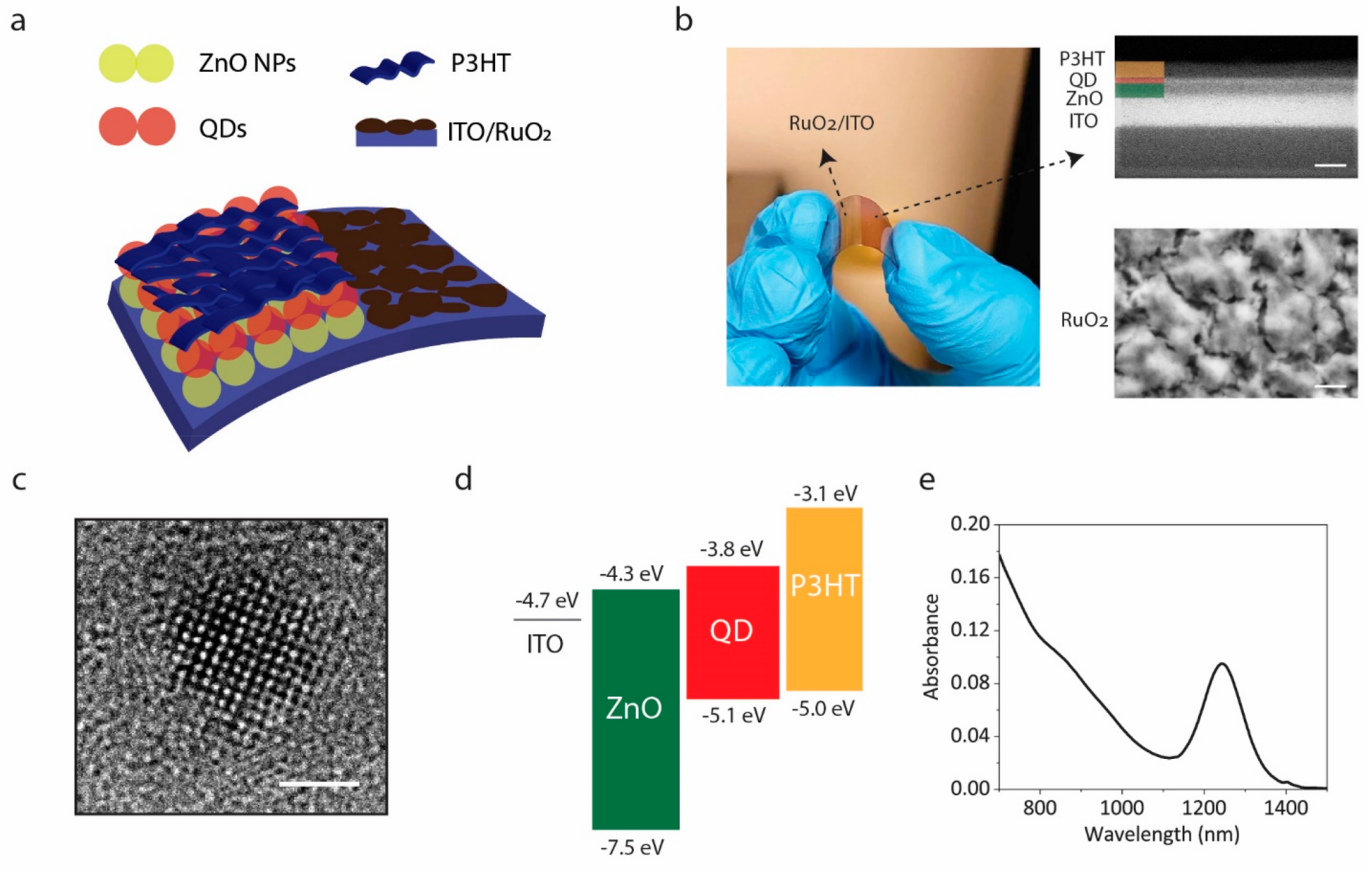
Biointerface design and properties. (a) Schematic of the multilayered biointerface architecture. (b) Left: Photograph of a typical device fabricated on a flexible PET substrate. Right: SEM image of QD-BI (top, scale bar is 100 nm) and RuO_2_ coating (bottom, scale bar is 200 nm). (c) HR-TEM image of the PbS QDs integrated into photovoltaic device. Scale bar is 2 nm. (d) Electronic energy levels of each layer and their alignment with respect to vacuum level. The levels were obtained from our previous studies.^27,28^ (e) Absorption spectrum of QDs between 700 and 1500 nm wavelengths. The device absorbance is shown in Figure S2.

The QD-based biointerface architecture consists of a ruthenium oxide (RuO_2_)-coated indium tin oxide (ITO) return electrode, a ZnO electron transport/hole-blocking layer, a NIR-absorbing PbS QD layer, and a poly(3-hexylthiophene-2,5-diyl) (P3HT) hole transport layer (Figure 1a). Thus, the device architecture consists of an active electrode (ZnO/PbS/P3HT/ITO) for photocurrent generation and a return electrode (RuO_2_/ITO), which completes the electrical path of the photocurrent. All the layers are solution-processed on an ITO/polyethylene terephthalate (PET) substrate, resulting in a flexible QD-based biointerface (QD-BI) (Figure 1b left). The cross-sectional scanning electron microscopy (SEM) image of QD-BI shows individual layer thicknesses as 50, 25, and 50 nm for ZnO, QD, and P3HT layers, respectively (Figure 1b top right). Together with the ITO back electrode (130 nm), the electronic layers of biointerfaces are 250 nm thick, which is advantageous for fabricating lightweight and flexible stimulation electrodes. Moreover, the surface SEM image of RuO_2_ coating demonstrates a porous film morphology, leading to a high electrochemical surface area/geometrical surface area (ESA/GSA) ratio that is favorable for obtaining a large interfacial capacitance.^25^ Transmission electron microscopy (TEM) analysis of QDs revealed the mean particle size as 3.6 ± 0.5 nm (Figure S1), and a high-resolution TEM (HR-TEM) image clearly displays the fine crystallinity of QD nanostructure (Figure 1c). Energy band alignment of the device architecture is favorable for separating the electron–hole pairs that are photogenerated at the QD layer (Figure 1d), while the 1.1 eV bandgap of the QDs provides NIR sensitivity to our biointerfaces, resulting in an absorption spectrum covering the NIR-I region (760–900 nm) and extending into the NIR-II region (1000–1700 nm) (Figure 1e).^26^ This provides our biointerfaces with a wide operation spectrum. However, as the absorption of light by water increases significantly beyond 900 nm, we use a photoexcitation wavelength of λ = 780 nm in our experiments.

**Figure 2:**
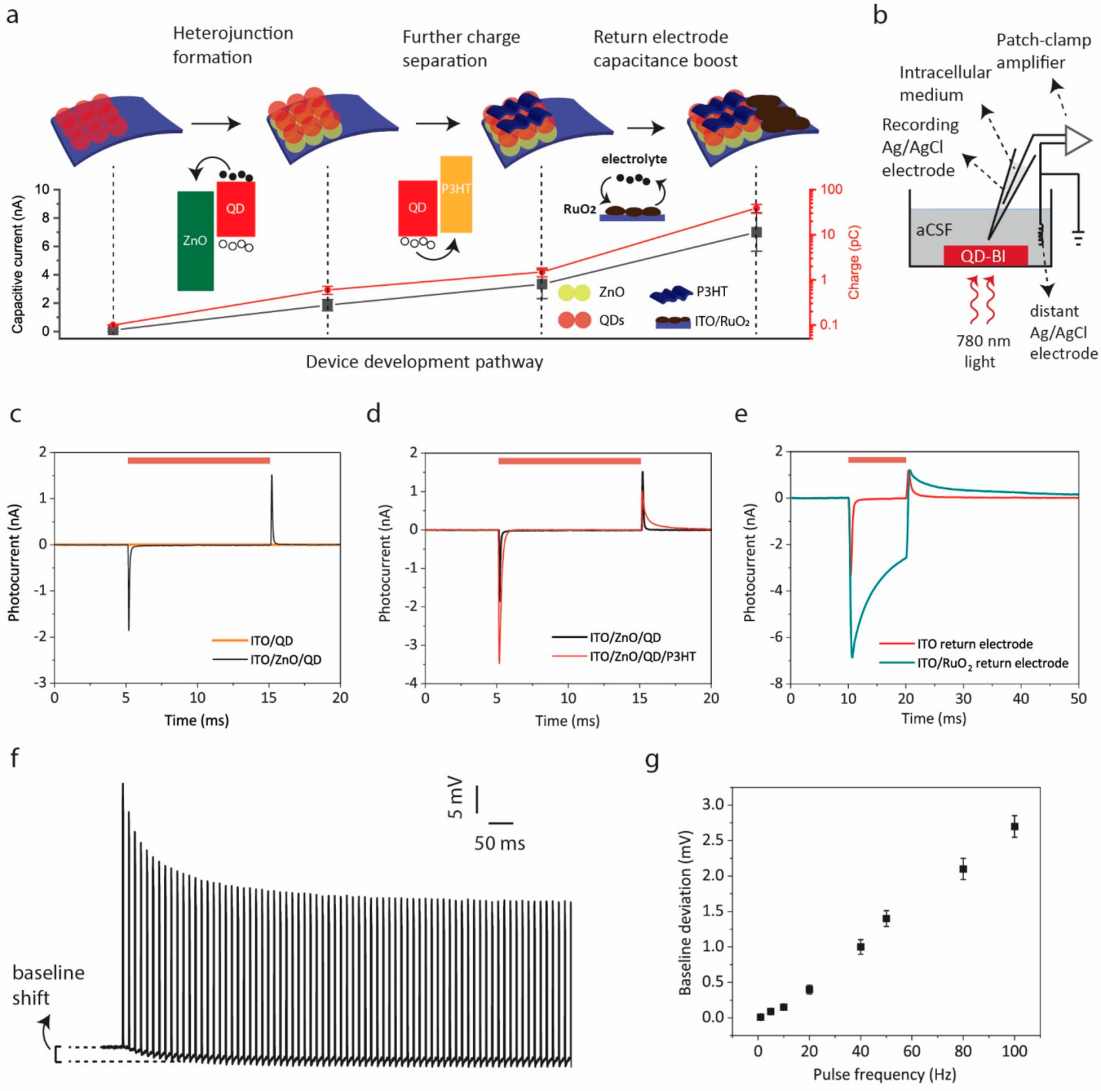
Photoelectrical characterization and device development. (a) A schematic presenting the origins of the device development pathway. Filled circles and empty circles represent electrons and holes, respectively. The plot below displays the evolution of capacitive onset current and photogenerated charge amplitude (mean ± s.d. for *N* = 6), which were measured with the setup described in panel b under 10 ms light pulses. Capacitive current stands for the peak photocurrent at the light onset.^32^ (b) Illustration of the open-circuit photocurrent/photovoltage measurement setup. A patch-clamp amplifier in voltage-clamp mode was used to record the photocurrents between the recording Ag/AgCl electrode and a distant Ag/AgCl reference electrode by positioning the patch-pipette close (<5 μm) to the device–electrolyte interface. The electrolyte is an artificial cerebrospinal fluid (aCSF), which mimics the in vivo extracellular medium of neurons (absorption spectrum of aCSF is provided in Figure S3). Effect of (c) ZnO, (d) P3HT, and (e) RuO_2_ layers on the photocurrent response of the biointerfaces. Red bars indicate light-on periods. (f) Photovoltage of QD-BI under 1 ms pulses applied at 100 Hz pulse frequency. (g) Deviation of the baseline photovoltage under 1 ms pulses for different pulse frequencies (mean ± s.d. for *N* = 6). For all measurements in this figure, optical power density was 5 mW mm^–2^ and patch-pipette resistance was 5 MΩ.

Each layer in the device architecture contributes to the photoelectrical performance of the biointerface, which are schematically summarized in Figure 2a. We quantified the effect of each step in the device development by measuring the interfacial photocurrent and photocharges generated at the device–electrolyte interface via a patch clamp setup (Figure 2b). The photoactive QD layer absorbs the incoming NIR photons and generates electron–hole pairs. To effectively separate these charge pairs, we integrated a ZnO NP layer between the ITO layer and the QD layer to form a charge separation heterojunction. Previous reports noted that air-exposed fabrication of the PbS QD layer leads to p-type doping,^29,30^ while ZnO is inherently an n-type material.^31^ This leads to an effective charge separation at the QD–ZnO interface by formation of an excitonic or depleted heterojunction,^31^ leading to the generation of capacitive response in the ITO/ZnO/PbS structure, while the ITO/PbS structure by itself has nearly zero photocurrent under NIR illumination (Figure 2c). Addition of a P3HT layer on top of the ITO/ZnO/PbS structure provides further improved charge separation due to its favorable highest occupied molecular orbital (HOMO) level for hole transfer. This leads to 2.1 ± 0.3 (mean ± s.d. for *N* = 6) times increase in the capacitive onset peak (Figure 2d). In addition to the improved capacitive response, we integrated a high-capacitance RuO_2_ layer to the return electrode to boost the charge injection density of the biointerfaces. Charge injection density increases by more than an order-of-magnitude with RuO_2_ integration (Figure 2e) because of the large interfacial capacitance of RuO_2_ resulting from fast and reversible redox reactions. Thus, the RuO_2_-integrated-ITO/ZnO/PbS/P3HT architecture yielded the champion photoelectrical performance in terms of capacitive response and charge injection density.

We also explored the suitability of QD-BI to high-frequency neurostimulation (on the order of tens of Hz). Because RuO_2_ coating on the return electrode increases the time constant, the decay of the photocurrent to its baseline value is slower after the light offset. This can cause charge accumulation at the electrode–electrolyte interface when high frequency pulses are applied and the charging at the interface would affect the resting potential of neurons that are cultured on QD-BI. We tested the deviation of photovoltage from its baseline under different pulse frequencies. Under 100 Hz stimulus with a 1 ms pulse width, the electrode–electrolyte interface rapidly charges at the beginning of the pulse train and charging is saturated after a few hundred milliseconds (Figure 2f). Consequently, the base photovoltage shifts by 2.7 ± 0.15 mV (mean ± s.d. for *N* = 6). We similarly quantified the amount of the baseline shift for different frequencies (Figure 2g), which reveals that the effect of charging at the electrolyte interface is marginal, i.e., less than a few millivolts. Hence, we expect no significant charging at the device–neuron interface during photostimulation experiments.

**Figure 3:**
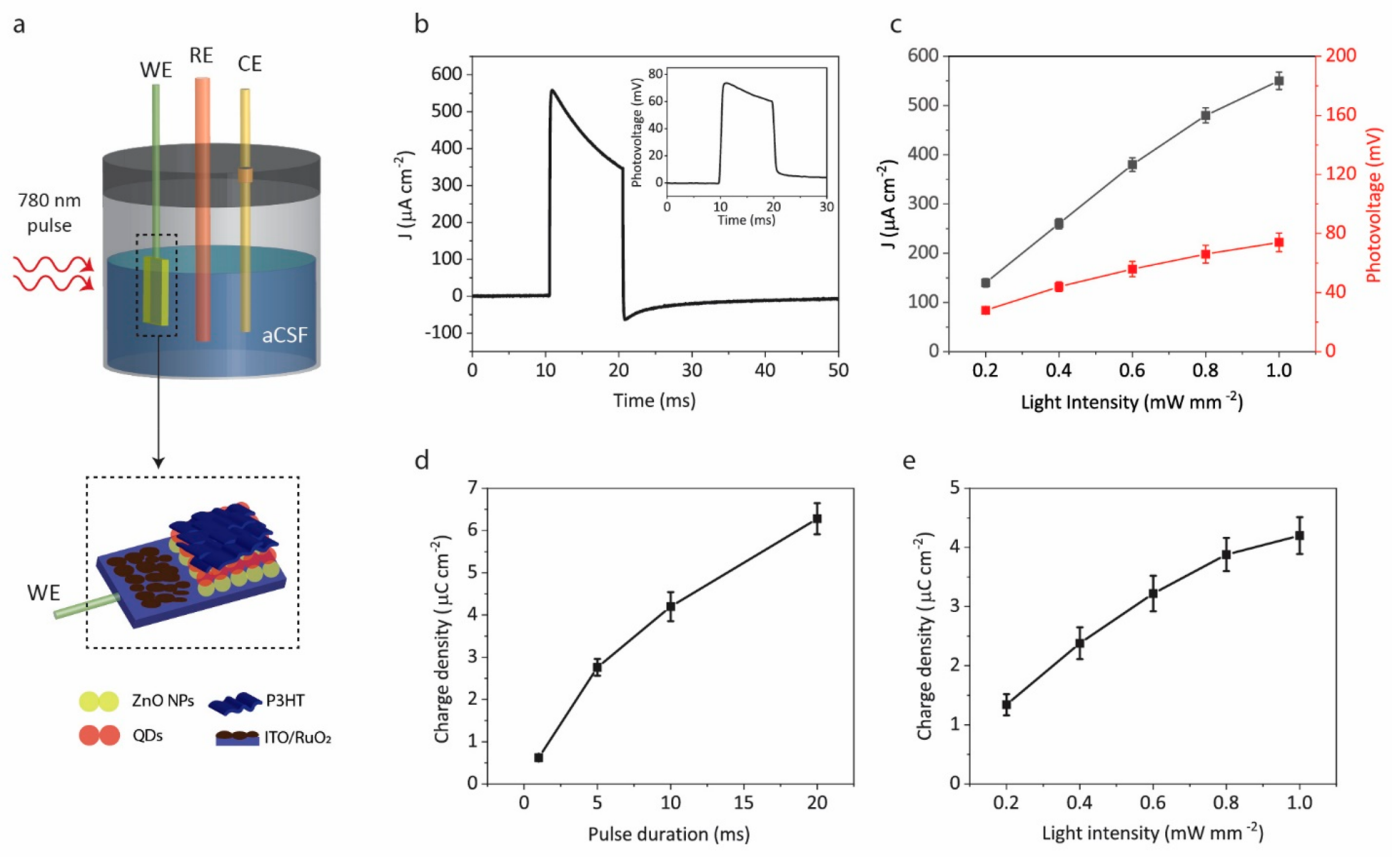
Current and charge density of the biointerfaces. (a) Three-electrode setup for characterization of short-circuit photoelectrical response of QD-BI with ITO return electrode as WE, platinum CE, and Ag/AgCl RE. Dashed area is zoomed view below for denoting the connection and device architecture. (b) Photocurrent response of QD-BI measured under a 780 nm light pulse with a 10 ms pulse width, 1 mW mm^–2^ light intensity, and 1 Hz pulse frequency. Inset shows the photovoltage under same conditions. (c) Photocurrent density and photovoltage of QD-BI as a function of light intensity (mean ± s.d. for *N* = 6). Photogenerated charge density of QD-BI as a function of (d) pulse duration and (e) light intensity (mean ± s.d. for *N* = 6). The light intensity was 1 mW mm^–2^ in panel d and pulse duration was 10 ms in panel e.

Short-circuit photoelectrical response of the biointerfaces is useful for evaluating the photoconversion efficiency of the electrodes. We measured the short-circuit photocurrent of QD-BI via a conventional three-electrode setup. The working electrode (WE) is connected to the return electrode of QD-BI, while the reference electrode (RE) and counter electrode (CE) are floating in the ionic medium of the artificial cerebrospinal fluid (aCSF) (Figure 3a). Application of 10 ms pulses of 780 nm with a 1 mW mm^–2^ optical power density resulted in a 550 μA cm^–2^ peak current density for QD-BI (Figure 3b). This corresponds to 5.5 mA/W responsivity. For a comparison, we checked the current density of QD-BI under 940 nm light as well and observed that the current density is higher for 780 nm (Figure S4), which we ascribe to the elevated absorbance of aCSF for wavelengths above 900 nm (Figure S3). Figure 3c shows the photocurrent and photovoltage as a function of light intensity, which has an almost linear dependence between photoresponse and incident power, indicating a single-photon-absorption induced photocurrent. The photocurrent in Figure 3b decays slowly because of the increased time constant by the integration of RuO_2_ to the return electrode and this significantly improves the charge injection density of QD-BI required for efficacious stimulation of neurons. We quantified the charge injection performance of QD-BI by calculating the areas under the photocurrent–time traces for different pulse widths (Figure 3d) and for different light intensities (Figure 3e). Accordingly, QD-BI delivers more than a 5 μC cm^–2^ charge for 20 ms pulses with a 1 mW mm^–2^ light intensity, which is in the range of threshold charge levels for stimulation of different structures like the optic nerve, auditory nerve, and subthalamic nucleus.^25^ Because of the experimental configuration in this measurement, the maximum light intensity is 1 mW mm^–2^, while in the photostimulation experiment setup, the light intensity can reach up to 7 mW mm^–2^, which means that the charge density will be further increased. Favorably, these intensities are below the ocular safety limits for pulse durations between 0.1 and 20 ms for stimulus frequencies of 1, 5, 10, and 20 Hz (Figure S5).

### Stability and Biocompatibility of QD-BI

**Figure 4:**
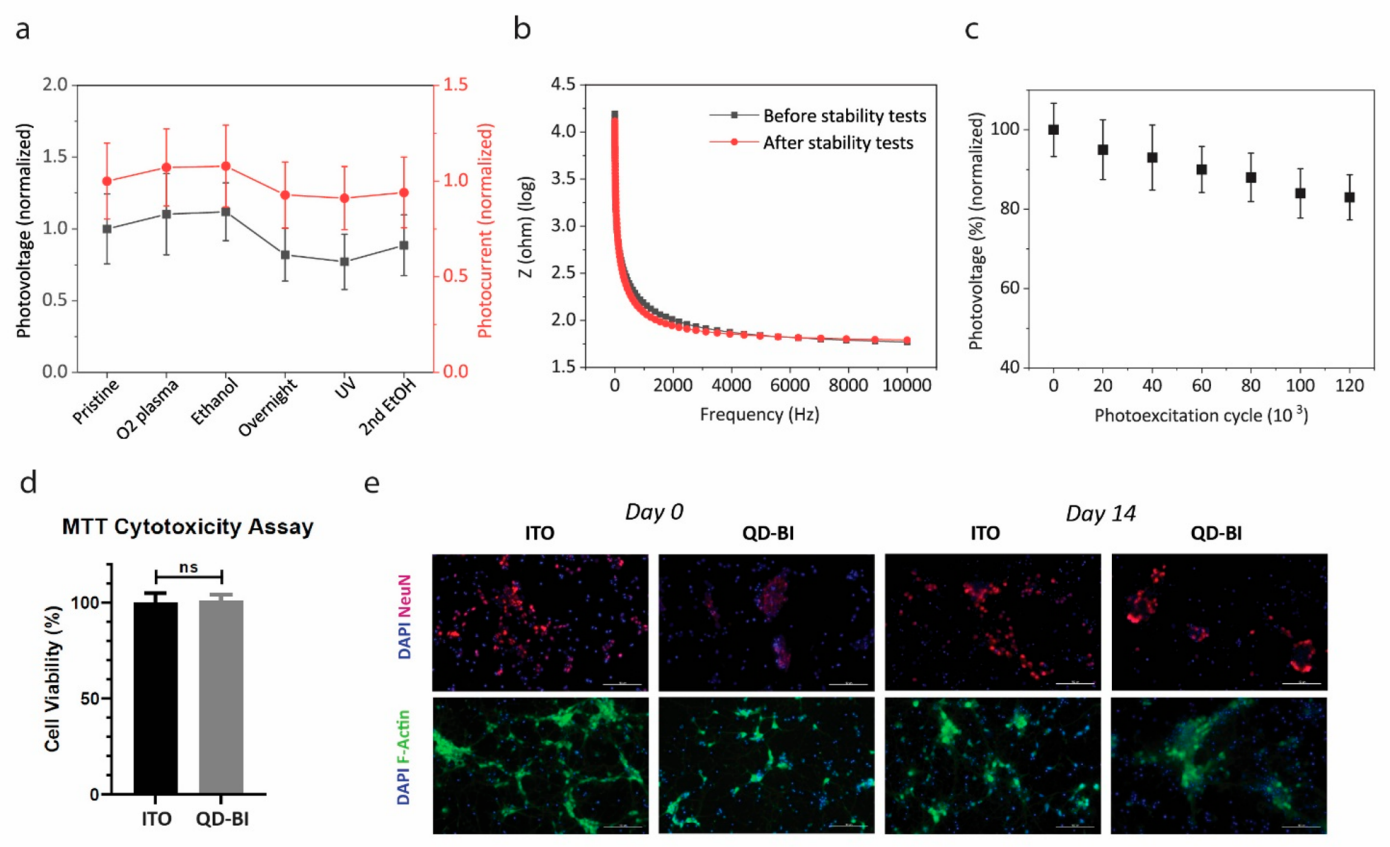
Stability and biocompatibility tests. (a) Photovoltage and photocurrent peak and of QD-BI under the accelerated stress test (mean ± s.d. for *N* = 4). Measurements of the two parameters were taken after each step, i.e., O_2_ plasma sterilization, EtOH (ethanol rinsing), Overnight (overnight incubation in cell culture medium), UV sterilization, and second ethanol rinsing. (b) Impedance measurement of QD-BI before and after the accelerated stress test between 1 and 10 000 Hz frequencies (traces represent the average of four different samples). (c) Photovoltage peak of QD-BI under 100 Hz photoexcitation for a 20 min (corresponding to 120 000 cycles) photostability test (mean ± s.d. for *N* = 4). Illumination: 100 Hz stimulus frequency, 5 ms pulse width, 7 mW mm^–2^ optical power density. (d) MTT cytotoxicity analysis of primary hippocampal neurons cultured on QD-BI and ITO control samples (mean ± sem for *N* = 4). The level of significance was calculated using an unpaired, two-tailed *t* test; *p < 0.05 was evaluated as statistically significant. (e) Immunofluorescence images of primary hippocampal neurons cultured on QD-BI and ITO control samples at day 0 and day 14 of incubation (each image is the average of four different images taken from four different areas). Primary hippocampal neurons were costained with DAPI (blue), Anti-NeuN (red), and Anti-F-actin (green). Scale bar: 100 μm.

To evaluate the photoelectrical stability of QD-BI, we measured the photovoltage and photocurrent of our devices after subjecting them to several sterilization procedures. This accelerated stress test gives information about the stability of devices under standard sterilization steps. Sequential application of O_2_ plasma sterilization, ethanol rinsing, overnight incubation in cell culture medium, UV sterilization, and second ethanol rinsing steps did not lead to a significant change in the photovoltage peak and photocurrent of QD-BI (Figure 4a). We also checked the impedance of pristine and sterilized electrodes via electrochemical impedance spectroscopy (EIS), which revealed that device impedance was not affected notably by the sequential sterilization procedure (Figure 4b), indicating that no considerable damage occurred during the accelerated stress test. The photostability of electrodes under repeated photoexcitation is also critical for anticipating the long-term operation of biointerfaces in a possible implant condition. The measurements of the photovoltage peak of QD-BI under a 100 Hz photoexcitation after 20 min, which corresponds to 120 000 photoexcitation cycle, showed that 82 ± 3% of the photovoltage peak was preserved after the photostability test (Figure 4c). For the origin of degradation, we consider that QDs are oxidizing due to repeated photoexcitation and being in an oxygen environment. According to the previous literature, rather than the intrinsic characteristics, the choice of ligand directly affects degradation processes in PbS QDs.^33^ Even though small oleic acid-capped PbS QDs are optically stable, the QDs used in this study with a first excitonic peak around 1.2 μm may not be perfectly stable in that sense, which can be either solved by decreasing the size of QDs or synthesizing QDs with enhanced Cl ions on the surface.^29,34^ Also, we do not observe a significant variation in the pH of the aCSF during repeated photoexcitation (Figure S6). Altogether, these experiments demonstrate that QD-BI retains its functionality in aCSF medium under different stress-inducing factors like sterilization tests and repeated photoexcitation.

We tested the viability of primary hippocampal neurons cultured on QD-BI via MTT cytotoxicity analysis to assess the biocompatibility of the biointerfaces. Cell viability of neurons cultured on QD-BI and ITO control substrates was compared after 48 h of incubation in the cell culture medium. Neurons cultured on QD-BI showed high cell viability and did not show any significant viability difference compared to the ones that were cultured on ITO control samples, indicating the low cytotoxicity of QD-BI for in vitro hippocampal neurons (Figure 4d). Moreover, immunofluorescence images of neurons on QD-BI and ITO control substrates taken at the 1st (day 0) and 14th day (day 14) of incubation demonstrated that neurons still survived and preserved their morphology on both QD-BI and ITO samples after 2 weeks of incubation (Figure 4e). Here, we consider that even though the heavy metal content of QDs is a possible source of toxicity, the biocompatible P3HT overcoating encapsulates the QDs and decreases the toxicity for the time period that we investigate the in vitro condition. Similarly, it was previously shown that encapsulation of QDs via a heavy-metal-free inorganic shell or organic coatings significantly suppresses the potential toxicity.^35,36^

### Photostimulation of Primary Neurons

**Figure 5:**
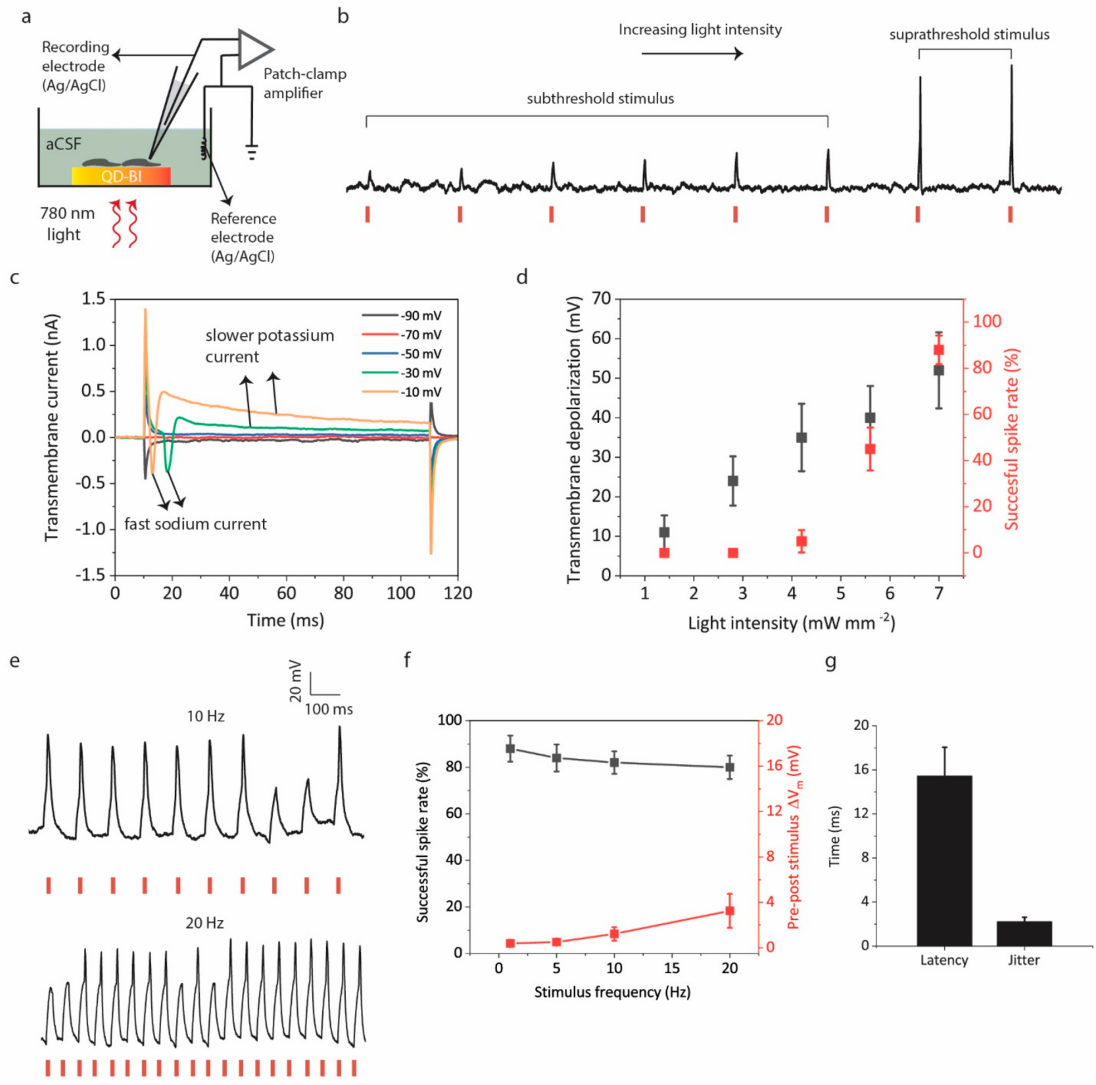
Light-induced neural stimulation. (a) Simplified schematic of intracellular recording setup using patch-clamp amplifier. Biointerfaces are electrically floating in extracellular solution of aCSF. (b) Current-clamp recordings of hippocampal neurons under gradually increased light intensity. The intensity in the beginning (4.2 mW mm^–2^) was increased by 0.4 mW mm^–2^ at each pulse. (c) Transmembrane current recording of neurons in whole-cell, voltage-clamp mode for different membrane holding potentials. (d) Dependence of transmembrane depolarization and spike success rate to the light intensity impinging on QD-BI (mean ± s.d. for *N* = 4). (e) Current-clamp recordings of neurons under repeated photostimulus for 10 and 20 Hz frequencies under 7 mW mm^–2^, 20 ms pulses. The stimulation artifacts at the onset and offset of light is eliminated because of downsampling of current clamp data (Figure S7). (f) Dependence of spike success rate and Δ*V*_m_ (difference in the membrane potential before and after 1 min of stimulus) to photostimulus frequency under 7 mW mm^–2^, 20 ms pulses (mean ± s.d. for *N* = 4). (g) Mean latency of action potentials and jitter (standard deviation of latencies for all firing neurons) for 7 mW mm^–2^, 20 ms pulses (mean ± s.d. for *N* = 4 neurons).

Stable operation and biocompatibility of QD-BI together with the effective photoelectrical performance point out its potential for light-induced electrical neurostimulation. To validate this, we performed single-cell intracellular recording experiments with a patch-clamp setup in whole-cell configuration. Primary hippocampal neurons were cultured on QD-BI and their light-induced transmembrane potential (defined as the intracellular membrane potential with respect to a distant Ag/AgCl electrode) behaviors were recorded in a current-clamp mode under 780 nm pulsed excitation (Figure 5a).

Light intensity impinging on the biointerface directly affects the response of neurons to photoexcitation. For lower light intensities, neurons exhibit subthreshold membrane responses. After a certain intensity level, neurons start to fire action potentials because of the suprathreshold depolarizing effect of QD-BI (Figure 5b). To understand the required amount of membrane depolarization for observing suprathreshold membrane response, we examined the transmembrane current of neurons in the whole-cell mode while gradually increasing the membrane holding potential in the voltage-clamp mode. At a −70 mV holding voltage, the transmembrane current is almost zero. When we increase the holding potential by −10 mV steps, we start to observe a fast negative inward current at −30 mV. This negative current is representative of a fast sodium inward current observed during an action potential, which is followed by a slower outward potassium current (Figure 5c). This means that the depolarization of the transmembrane potential on the order of 40 mV is expected to elicit action potential firing. When we increase the holding potential to −10 mV, we again notice the rapid sodium inward current with a lower latency compared to a −30 mV holding potential as expected.

Next, we characterized the amount of transmembrane depolarization under different intensities and how these reflect into the ratio of successful/unsuccessful spikes of action potential for each intensity. To quantify the magnitude of depolarization without inducing action potentials, we blocked the voltage-gated sodium channels by adding 5 mM QX-314 chloride into the intracellular solution. We observe an almost linear dependence of the depolarization magnitude to the light intensity (Figure 5d). A 4.2 mW mm^–2^ light intensity generates a 35 ± 8.5 mV depolarization and a low (5 ± 4.5%) successful spike rate at 1 Hz with a 20 ms pulse duration because only a minor part of the pulses produces suprathreshold (greater than 40 mV) transmembrane depolarization. For a 5.6 mW mm^–2^ intensity, depolarization and spike rate increases to 40 ± 8.1 mV and 45 ± 9%, respectively. The spike rate jumps to 88 ± 6% for 7 mW mm^–2^ light intensity, meaning that the generated transmembrane depolarization (52 ± 9.2 mV) is sufficient for most of the pulses to elicit firing. QD-BI can also evoke reproducible action potentials for higher frequency stimuli such as 10 and 20 Hz as well (Figure 5e). The spike success rates for 1, 5, 10, and 20 Hz frequencies are all above 80%, indicating an efficient coupling of the photoresponse of biointerfaces to the neural membrane (Figure 5f). Moreover, after the application of photostimulus of 1, 5, 10, and 20 Hz frequencies for a duration of 1 min, there is only a marginal change (maximum of 3.2 ± 1.3 mV at 20 Hz) in the resting membrane potential of neurons (Figure 5f). Finally, we calculated the mean latency and jitter parameters for the successful spikes induced by QD-BI as 15.4 ± 2.4 ms and 2.2 ± 0.4 ms for 20 ms pulse-width photostimulus, respectively (Figure 5g).

## Conclusions

This study demonstrated a QD-based NIR-responsive photovoltaic biointerface. For operation in the tissue transparency window, we chose an NIR-absorbing QD and combined it with a ZnO NP layer to achieve an effective charge separation and capacitive photoresponse. Addition of a P3HT hole transport layer further improved the photoresponse by capturing holes, while RuO_2_ coating on the return electrode led to a large return electrode capacitance and high charge injection density. All these layers were coated via solution-processed techniques, indicating the simple and low-cost manufacturability of QD-based biointerfaces.

Analysis of the photocharge generation of QD-BI in the ionic medium showed that the dominant charge injection mechanism is capacitive at the electrode–electrolyte interface,^37^ which is a safe alternative to irreversible faradaic charge injection.^38^ Moreover, addition of RuO_2_ to the return electrode introduces fast and reversible faradaic processes at the RuO_2_–electrolyte interface, which are counterbalanced by the faradaic reactions occurring at the active electrode (ZnO/PbS/P3HT)–electrolyte interface. We did not observe any sign of presence of irreversible faradaic reactions during the photostimulation experiments in terms of pH variation, cell viability, and degradation of the biointerface.

Even though silicon photodiodes have been applied to NIR photovoltaic stimulation and showed high efficiency,^39,40^ their transition to flexible device architectures has not occurred yet. On one hand, nanomaterial forms of silicon have been integrated into flexible device structures, but their spectrum has remained in the visible region.^41^ On the other hand, although the potential of organic biointerfaces was revealed with a computational study for retinal implants,^8^ only one recent report experimentally demonstrated neuromodulation at the NIR.^10^

High absorption coefficient of the QDs^24^ and the effective device design of our biointerfaces resulted in photostimulation of neurons with light intensities below the ocular safety limits (Figure S2), which is critical for retinal prosthetic devices. The maximum permissible exposure values are higher for NIR light compared to visible light, which renders NIR-responsive biointerfaces more suitable for ophthalmic applications.^42^ Furthermore, the retinal receptors are responsive to the visible spectrum but not to NIR wavelengths, thus flexible NIR-responsive biointerfaces combined with smart goggles with NIR-projectors have high potential to be used to recover vision against retinal degeneration diseases. In addition, NIR light can penetrate a few centimeters deep into the brain and such NIR-responsive biointerfaces can be also used for brain stimulation by engineering them according to the required tissue mechanics.

In conclusion, we presented a QD-based biointerface that can trigger NIR-light-induced action potentials on hippocampal neurons in a tissue transparency window. Favorably, biointerfaces can be fabricated on a flexible substrate using simple solution-processed methods, and an ultrathin QD layer in a well-designed photovoltaic architecture results in efficient capacitive photocurrent generation that leads to temporally precise and reproducible photostimulation of neurons. QDs with their exceptional optoelectronic and bioconjugation properties hold high promise for next-generation neural interfaces.

## Experimental Section

### Device Fabrication

ITO/PET with a surface resistivity of 60 Ω sq^–1^ (Sigma-Aldrich), PbS core-type quantum dots (Sigma-Aldrich, λ_em_ = 1400 nm, 10 mg mL^–1^ in toluene), P3HT with a regioregularity of 95.7% and a molecular weight of 57 467 g mol^–1^ (Ossila), ruthenium(III) chloride hydrate (RuCl_3_·*x*H_2_O) with a molecular weight of 207.43 g mol^–1^ (Sigma-Aldrich), zinc acetate dehydrate (Zn(CH_3_CO_2_)_2_·2H_2_O) (Sigma-Aldrich), 2-methoxyethanol (C_3_H_5_O_2_) (Sigma-Aldrich), ethanolamine (HOCH_2_CH_2_NH_2_) (Sigma-Aldrich), and 1,2-dichlorobenzene (C_6_H_4_Cl_2_) were used in the fabrication. Although we used 780 nm excitation in our study, we used a red-shifted QD (λ_em_ = 1400 nm) because the absorbance of the QDs increases toward 780 nm due to the additional electronic transitions occurring between the conduction and valence bands.

For cleaning, substrates were consecutively sonicated in a detergent solution, deionized water, acetone, and isopropyl alcohol for 15 min. Dried substrates were subjected to UV ozone treatment for 20 min. Then, a ZnO precursor sol–gel solution, which consisted of 219.3 mg of zinc acetate dehydrate (Zn(CH_3_CO_2_)_2_·2H_2_O), 2 mL of 2-methoxyethanol (C_3_H_5_O_2_), and 73 mg of ethanolamine (HOCH_2_CH_2_NH_2_), was spin-coated at 2000 rpm and annealed at 200 °C for 20 min. The PbS QD solution was spin coated at 2000 rpm and annealed at 100 °C for 15 min. Then a 20 mg mL^–1^ P3HT solution in 1,2-dichlorobenzene was spin coated at 2000 rpm and annealed at 150 °C for 15 min. RuO_2_ was coated via 60-cycle electrochemical deposition from a 0.01 M RuCl_3_·*x*H_2_O solution as described in a previous study.^43^

### Photoelectrical Characterization

An EPC 800 Heka Elektronik patch-clamp amplifier was used for recording open-circuit photoelectrical parameters. Extracellular medium (artificial cerebrospinal fluid (aCSF)) was prepared by mixing 10 mM 4-(2-hydroxyethyl)-1-piperazineethanesulfonic acid (HEPES), 10 mM glucose, 2 mM CaCl_2_, 140 mM NaCl, 1 mM MgCl_2_, 3 mM KCl, and a stoichiometric amount of NaOH to adjust the pH to 7.4, in distilled water. The intracellular medium was prepared by mixing 140 mM KCl, 2 mM MgCl_2_, 10 mM HEPES, 10 mM ethylene glycol-bis(β-aminoethyl ether)-*N*,*N*,*N*′,*N*′-tetraacetic acid (EGTA), 2 mM Mg-ATP, and a stoichiometric amount of KOH to adjust the pH to 7.2–7.3, in distilled water. Biointerfaces were left floating in aCSF without any wire connection. Patch pipettes were filled with the intracellular medium.

An Autolab Potentiostat Galvanostat PGSTAT302N (Metrohm, Netherlands) was used for recording short-circuit photocurrent/photovoltage in a three-electrode configuration. The back electrode of thin film samples was connected to the working electrode. The Ag/AgCl reference electrode and platinum counter electrodes were used. Measurements were performed in ionic medium aCSF.

Thorlabs M780LP1 LED was used as the illumination source. A Thorlabs DC2200 High-Power 1-Channel LED Driver was used to adjust the pulse widths and light intensities. Optical powers were measured via a Newport 843-R power meter.

### Sterilization Procedures

O_2_ plasma sterilization was applied for 5 min. First and second ethanol rinsings were performed three times. Overnight sterilization was incubation of the devices in the cell culture medium at 37 °C for 24 h.

### Electrochemical Impedance Measurement

EIS was performed using an Autolab Potentiostat Galvanostat PGSTAT302N (Metrohm, Netherlands) in the same three-electrode configuration described in the Photoelectrical Characterization. The frequency range was 1 Hz–10 kHz in the EIS measurement and a 10 mV (RMS) AC voltage was applied. Measurements were taken in ionic medium aCSF.

### Primary Neuron Isolation

All experimental procedures were approved by the Institutional Animal Care and Use Committees of Koç University (Approval No: 2021.HADYEK.022) according to Directive 2010/63/EU of the European Parliament and of the Council on the Protection of Animals Used for Scientific Purposes. Procedures were carried out by responsible veterinarian and certified researchers for animal experiments. Primary hippocampal neuron isolation and culture protocols were performed according to our previous studies.^23,44^ Hippocampus of E15-E17 Wistar Albino rat embryos were isolated and placed immediately in ice-cold Hank’s Balanced Salt Solution (HBSS, Thermo Fisher Scientific, MA, USA). Enzymatic digestion of the hippocampus was performed with incubation in %0.25 Trypsin-EDTA solution (Thermo Fisher Scientific, MA, USA) with 2% DNase-I supplement (NeoFroxx, Einhausen, Germany) for 20 min in a 37 °C incubator. After digestion, the cells were centrifuged, and the supernatant was changed with Dulbecco’s Modified Eagle Medium/Nutrient Mixture F-12 (DMEM/F12 Thermo Fisher Scientific, MA, USA) supplemented with %10 fetal bovine serum (FBS, heat inactivated, GE Healthcare, IL, USA) and 1% penicillin/streptomycin (Thermo Fisher Scientific, MA, USA). DMEM/F12 media was discarded and Neurobasal Medium (NBM, Thermo Fisher Scientific, MA, USA) supplemented with B27, l-glutamine, β-mercaptoethanol, glutamate (Thermo Fisher Scientific, MA, USA) was added to the cell pellet. The cells were triturated and passed through a 70 μm cell strainer. The homogeneous cell solution was seeded in poly-d-lysine (PDL, Sigma-Aldrich, MO, USA) coated substrates. After 3 days of incubation of cells on substrates, the media of the cells were changed with NBM supplemented with cytosine arabinoside (Sigma-Aldrich, MO, USA) to inhibit growth of glial cells. After 24 h of incubation with cytosine arabinoside, the media were refreshed with NBM and primary hippocampal neurons on the substrates were cultured for further experiments.

### Biocompatibility Assay

Cell viability of primary hippocampal neurons on the biointerfaces was checked with a MTT assay according to our previous studies.^23,44^ Briefly, the biointerface devices were sterilized by 70% ethanol and UV irradiation for 30 min. Biointerfaces were placed in the 6-well plates. Primary hippocampal neurons were seeded on the substrates as 5 × 10^5^ cells per sample and cultured with in Neurobasal Medium (NBM, Thermo Fisher Scientific, MA, USA) supplemented with B27, l-glutamine, β-mercaptoethanol, and glutamate (Thermo Fisher Scientific, MA, USA) at 37 °C with 5% CO_2_. After 48 h of incubation, the cell media were replaced with 1 mL of MTT solution (5 mg/mL in PBS, pH = 7.4) and 4 mL of a NBM mixture per well, and the cells were incubated at 37 °C for 4 h. After 4 h of incubation, samples were transferred to a new 6-well plate, and a 1:1 mixture of DMSO and ethanol was added on the wells to dissolve the formazan crystals. The solution was transferred to a 96-well plate and the absorbance was measured at at 570 nm light with Synergy H1Microplate Reader (Bio-Tek Instruments). The relative cell viability was calculated as percentage of cell viability = (OD_sample_/OD_control_) × 100.

### Immunofluorescence Staining and Imaging

Primary hippocampal neurons (5 × 10^5^ cells per sample) were cultured as explained above on the ITO control, and the biointerface substrates were then allowed for Day 0 and Day 14 growth in an appropriate culture condition. Neurons on the substrates were fixed with 4% paraformaldehyde on Day 0 and Day 14 and washed three times with PBS-T (Phosphate Buffered Saline, 0.1% Triton X-100). Cells were blocked in a superblock solution. After the blocking treatment, cells on the substrates were incubated with rabbit anti-NeuN antibody (ab177487, Abcam, Cambridge, UK) overnight at 4 °C for neuron characterization, and washed three times with PBS-T. Then, samples were incubated with goat antirabbit IgG H&L Alexa Fluor 555 (4413, Cell Signaling Technology, MA, USA) for 90 min at 37 °C. For visualization of the cytoskeleton, primary neuron samples also were stained with a fluorescein isothiocyanate-conjugated phalloidin antibody (Sigma-Aldrich, P5282) for 90 min at 37 °C. All samples were washed three times with PBS-T, then mounted with a DAPI supplemented mounting medium (ab104139, Abcam, Cambridge, UK) to observe nuclei. Immunofluorescence imaging was performed with an inverted fluorescence microscope (Axio Observer Z1, ZEISS, Oberkochen, Germany).

### Electrophysiology Recordings

An EPC 800 Heka Elektronik patch-clamp amplifier was used for recording electrical activity of hippocampal neurons that were cultured on biointerfaces. The current-clamp recordings for transmembrane voltage and voltage-clamp recordings for transmembrane current measurements were performed in whole-cell configuration. No wire was connected to the biointerfaces. aCSF was used as the extracellular medium. The patch-pipette resistance of 5–8 MΩ was used for the recordings. Patch pipettes were filled with the intracellular medium as described above. For blocking the voltage-gated sodium channels, 5 mM QX-314 chloride was added into the intracellular medium. For the statistical analysis of action potentials, the current clamp data was downsampled without causing changes in the properties of action potentials to conduct the analysis with a feasible computational complexity. A digital camera integrated with the Olympus T2 upright microscope was used for monitoring the neurons and the movement of the patch pipette. Biointerfaces were illuminated from the bottom using M780LP1 Thorlabs LED driven by Thorlabs DC2200 LED driver.

### Optical Safety Considerations

The maximum permissible radiant power (MP) that can be chronically delivered to the retina was calculated according to the ocular safety standards.^42^ The photochemical limit does not apply in the NIR region and the equation for photothermal and photoacoustic limit is *MP* = 6.93 × 10^–5^*C*_T_*C*_E_ P^–1^. *C*_T_ = 10^0.002 (λ-700)^ = 1.445 for λ = 780 nm. *C*_E_ was taken as 29.3 W mm^–2^ considering a retinal spot size larger than 1.7 mm in diameter in accordance with a previous study.^40^ The equation for the single-pulse limit for the pulse-widths between 0.05 and 70 ms is given as *MP*_single_ = 6.93 × 10^–4^*C*_T_*C*_E_*t*^–0.25^. These equations give average irradiance limits and the peak irradiance limits can be calculated from *MP*_peak_ = *MP*_avg_/(*t*×*f*), where *t* is pulse duration and *f* is pulse frequency.

## Supporting Information Available

TEM analysis of PbS QDs, absorbance of the biointerface, absorbance of aCSF, photocurrent measurement under 940 nm excitation, calculated maximum permissible exposure limits, aCSF pH measurement under repeated photoexcitation, and elimination of capacitive artifacts (PDF)
