# Supplementary material for: Electrical Stimulation of Neurons with Quantum Dots via Near-Infrared Light
Source: ACS Nano. 2022 May 2;16(5):8233–43. doi: 10.1021/acsnano.2c01989 (PMC9134491; doi:10.1021/acsnano.2c01989)
Supplement: Supplementary file 1 — nn2c01989_si_001.pdf [file nn2c01989_si_001.pdf]

Supporting Information

Electrical Stimulation of Neurons with Quantum Dots via Near-infrared Light

Onuralp Karatum[1], Humeyra Nur Kaleli[2], Guncem Ozgun Eren[3], Afsun Sahin[2, 4], Sedat Nizamoglu[1, 3, *]

[1] Department of Electrical and Electronics Engineering, Koc University, Istanbul 34450, Turkey
[2] Research Center for Translational Medicine, Koc University, Istanbul 34450, Turkey
[3] Department of Biomedical Science and Engineering, Koc University, Istanbul 34450, Turkey
[4] Department of Ophthalmology, Medical School, Koc University, Istanbul 34450, Turkey

*** Correspondence:**
Sedat Nizamoglu
snizamoglu@ku.edu.tr

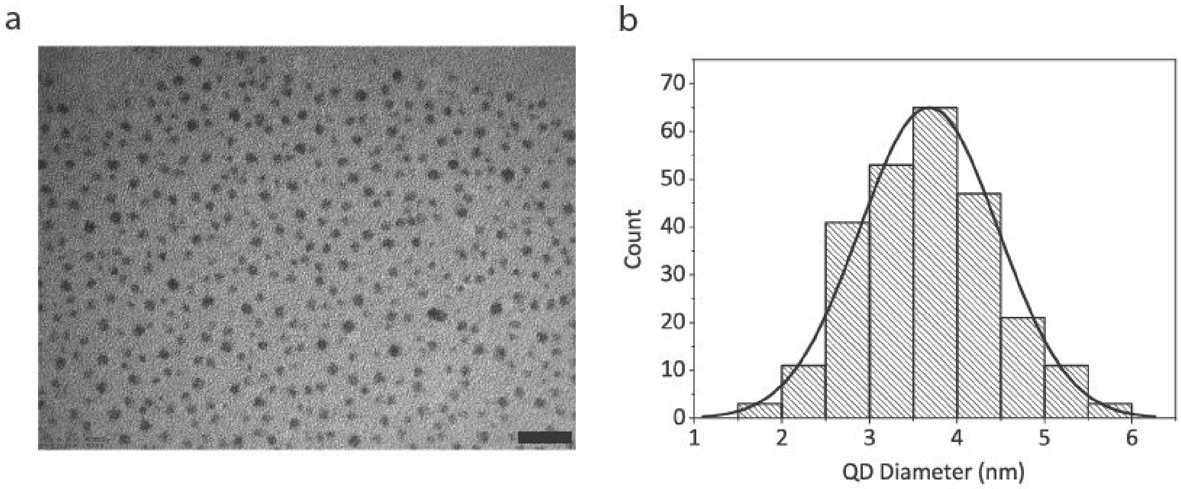

Figure S1: a) Transmission electron microscopy (TEM) image of PbS QDs (scale bar is 10 nm), and **b)** the corresponding size distribution (N > 250).

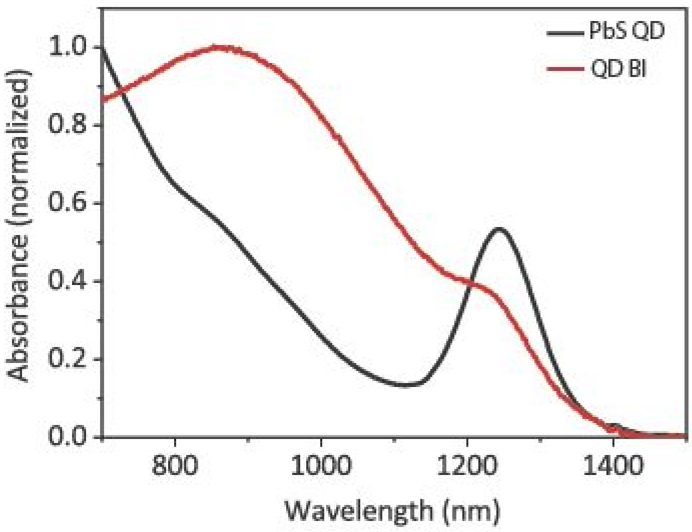

Figure S2: Absorbance of PbS QDs in solution and absorbance of the biointerface (QD BI). Normalized absorbance was provided for better comparison.

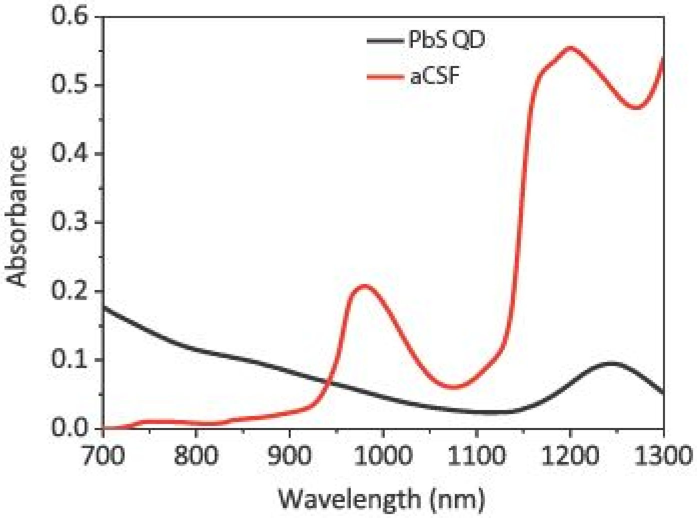

Figure S3: Absorbance of PbS QD and aCSF.

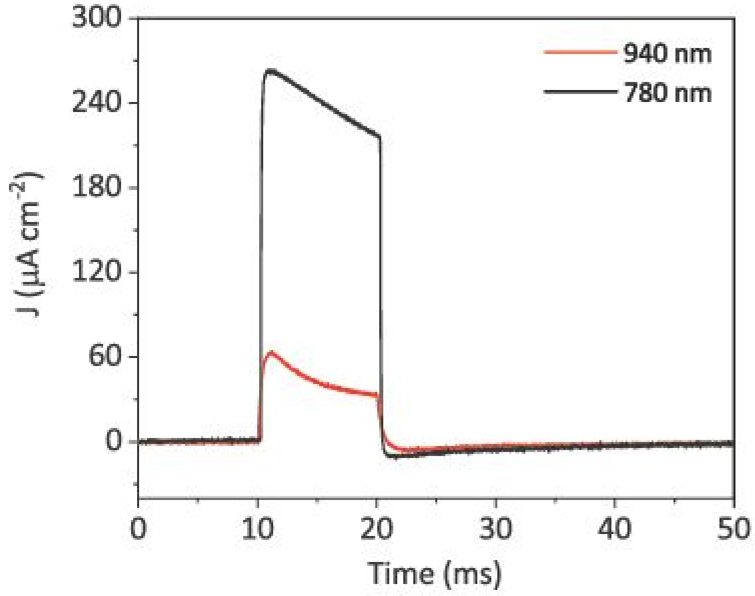

Figure S4: Photocurrent density of the biointerface under 780 nm (black), and 940 nm (red) photoexcitation. 10 ms pulse was applied at t = 10 ms. Light intensity was 0.4 mW mm^{-2} in both cases.

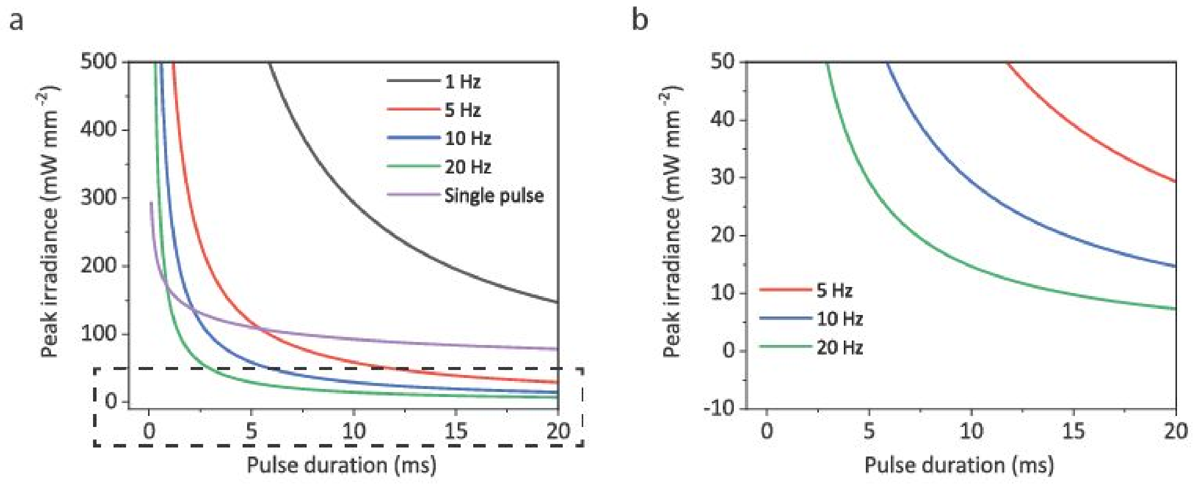

Figure S5. a) Maximum permissible exposure limits calculated according to Ocular Safety Standards for 780 nm pulsed light at different pulse-widths and pulse frequencies. **b)** Zoomed view of the dashed rectangle in (a).

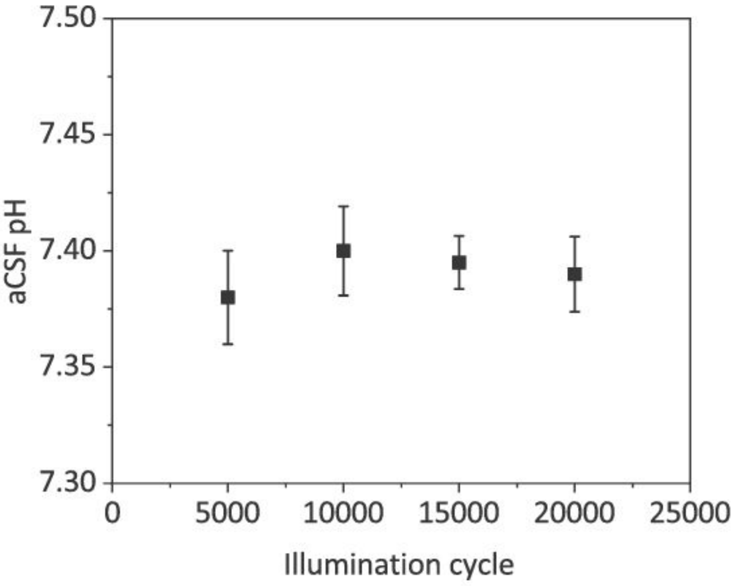

Figure S6. Variation of extracellular medium (aCSF) pH during repeated photoexcitation of quantum dot biointerfaces under 780 nm, 7 mW mm^{-2}, 10 ms pulse-width, 5 Hz photoexcitation (mean ± s.d. for N = 4).

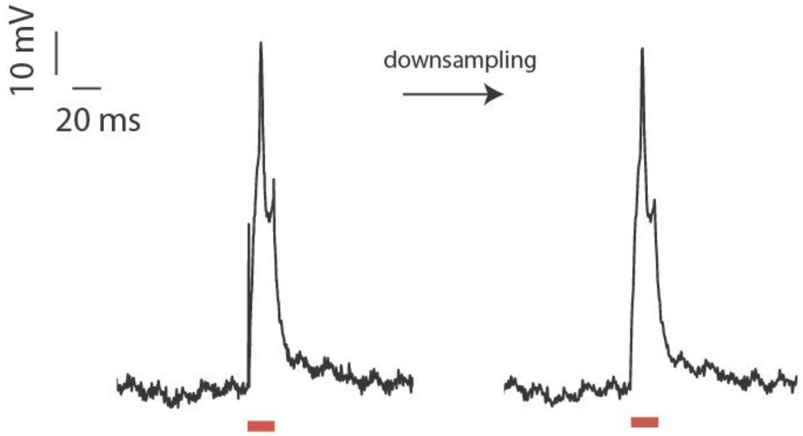

Figure S7. The elimination of capacitive artefacts at the light onset and offset because of downsampling of current clamp data. The properties of action potentials (e.g., threshold, latency, jitter) were preserved. Red bars indicate light on periods.
